# Balance of care activity after EMA recommendation for *DPYD* gene testing in Galicia

**DOI:** 10.3389/fphar.2025.1523536

**Published:** 2025-03-28

**Authors:** Almudena Gil-Rodríguez, Sheila Recarey-Rama, Ana Rodríguez-Viyuela, Francisco Barros, Angel Carracedo, Olalla Maroñas

**Affiliations:** ^1^ Pharmacogenomics and drug discovery (GenDeM), Health Research Institute of Santiago de Compostela (IDIS), Santiago de Compostela, Spain; ^2^ Genomics Medicine Group, CIMUS, University of Santiago de Compostela, Santiago de Compostela, Spain; ^3^ Genetics group, Health Research Institute of Santiago de Compostela (IDIS), Santiago de Compostela, Spain; ^4^ Center for Biomedical Network Research on Rare Diseases (CIBERER), Instituto de Salud Carlos III, Madrid, Spain; ^5^ Galician Public Foundation of Genomic Medicine (FPGMX), Galician Healthcare Service (SERGAS), Santiago de Compostela, Spain

**Keywords:** fluoropyrimidines, DPYD, implementation, pharmacogenetics, adverse-drugreaction, EMA, AEMPS

## Abstract

**Introduction:**

Since April 2020, pretherapeutic screening for accessing the deficiency of the DPD enzyme by genotyping the dihydropyrimidine dehydrogenase gene (*DPYD*) is required by the European Medicine Agency (EMA) prior to the administration of fluoropyrimidine-based chemotherapy. In May 2020, the Spanish Drug and Medical Devices Agency (AEMPS) published an informative note highlighting the importance of *DPYD* analysis prior fluoropyrimidines derivatives administration to prevent the development of severe adverse drug reactions (ADRs). The publication of these recommendations marked a turning point in the daily routine in many pharmacogenetics laboratories in Spain. This article aims to illustrate the current state of the *DPYD* testing in the reference genomic medicine center in Galicia, 4 years after the EMA’s updated recommendations.

**Methods:**

The Pharmacogenetics Unit in the reference genomic medicine center conducted genotyping of the four *DPYD* variants recommended by regulatory agencies that oncologists can adjust fluoropyrimidine treatment based on *DPYD* genotype results.

**Results:**

Between 1 June 2020 to 1 May 2024, both included, a total of 2,798 *DPYD* requests were analyzed. *DPYD* genotyping results revealed a 3.15% prevalence of heterozygosity for at least one of the four *DPYD* variants, being rs56038477 the most prevalent variant (1.31%).

**Conclusion:**

This study addresses the importance of the *DPYD* analysis implementation in clinical practice after the changes in EMA and AEMPs recommendations which has led to a significant increase in *DPYD* genotyping requests. This highlights the significance of preemptive genotyping for accurately adjusting fluoropyrimidines doses before initiating treatment.

## 1 Introduction

It is well known that fluoropyrimidines, 5-fluorouracil (5-FU) and its prodrugs, capecitabine and tegafur, are a group of cytostatic drugs used in the treatment of cancers, particularly related to the gastrointestinal tract (such as colorectal and gastric cancers), as well as head and neck and breast cancer (Simões et al., 2020). Although the efficacy of fluoropyrimidines has been demonstrated in numerous studies, these drugs are not exempt from side effects including diarrhea, vomits, mucositis, myelosuppression or hematological toxicity (Froehlich et al., 2015; Henricks et al., 2018). The therapeutical effect of fluoropyrimidines is based on the ability of 5-FU to act as an antimetabolite of uracil. The 5-FU is metabolized by an enzyme called dihydropyrimidine dehydrogenase (DPD), which is encoded by the *DPYD* gene. More than 80% of the dose is metabolized by the DPD enzyme, making this step the rate-limiting one in the metabolism of 5-FU^1^ (Diasio and Harris, 1989; van Kuilenburg et al., 2003; García-Alfonso et al., 2022). Adverse drug reactions (ADRs) are the result of decreased DPD activity due to slower degradation of 5-FU, resulting in high exposure of the drug and its cytotoxic metabolites (Longley et al., 2003; Lee et al., 2014; Simões et al., 2020).

*DPYD* is a highly polymorphic gene and specific polymorphisms can result in reduced or null activity of the resulting DPD enzyme. Patients with null or decreased DPD activity are at risk of developing ADRs, with higher severity in patients carrying null activity variants. It has been estimated that approximately 35% of the world’s population have at least one allele with reduced activity, while less than 1% carry two alleles of null activity. However, these percentages vary among populations^2^. In 2018, a prospective multicenter study demonstrated the feasibility of *DPYD* analysis in routine clinical practice and showed that *DPYD* genotype-based dose reductions improve patient safety of fluoropyrimidines. The study proposed four variants of *DPYD* which should be tested before treatment with fluoropyrimidines. These variants were rs3918290 (c.1905+1G>A), rs55886062 (c.1679T>G), rs56038477 (c.1236G>A) and rs67376798 (c.2846A>T), which have been widely reported in the literature to be strongly associated with the development of fluoropyrimidines toxicity (Lee et al., 2014; Toffoli et al., 2015; Meulendijks et al., 2016; Henricks et al., 2018; Madi et al., 2018). Although these polymorphisms appear at very low frequencies in populations, their presence significantly increases the toxicity associated with chemotherapy. Manifold studies have demonstrated the advantages of analyzing these *DPYD* variants before starting fluoropyrimidine therapy, as it leads to improved drug responses and reduced healthcare costs (Cortejoso et al., 2016; Henricks et al., 2017; van der Wouden et al., 2022; Brooks et al., 2022).

Alongside regulatory agencies, pharmacogenetics consortia, such as the Dutch Pharmacogenetics Working Group (DPWG) since 2011 and the Clinical Pharmacogenetics Implementation Consortium (CPIC^®^) since 2013 (Amstutz et al., 2018; Lunenburg et al., 2020), have already established dosage recommendations based on the *DPYD* genotype. New evidence published in the last years (Henricks et al., 2015, 2018; 2019) enable the EMA to change its initial recommendations for *DPYD* analysis as an actionable into mandatory in April 2020 (de With et al., 2023; Whirl-Carrillo et al., 2021). One month later, in May 2020, a specific guideline was developed by the Spanish Drug and Medical Devices Agency (AEMPS) which issued an alert on the necessity of analyzing *DPYD* before administering fluoropyrimidines^3^ ^,^ ^4^. These changes in recommendations have resulted in a surge in the daily number of *DPYD* analyses conducted in hospital laboratories. Furthermore, as the incidence of colorectal cancer continues to rise, with 42,721 new cases reported in Spain in 2023 (estimated by Spanish Society of Medical Oncology (SEOM)^5^) it is expected that the number of *DPYD* test will increase. In the case of Galicia, a report from the Spanish Association Against Cancer (AECC) in 2023 estimated 2,802 cases of CRC, accounting for 14.65% of the diagnosed cancer cases in this region^6^.

The main objectives of the present article are to illustrate the exponential rise in the number of *DPYD* tests conducted in Galicia (Spain) over 4 years following the change in recommendations, as well as to present the percentage of patients who have benefit from specific recommendations for being carriers of at least one decreased or null variant.

## 2 Methodology

### 2.1 Analysis center and healthcare system

The reference genomic medicine center in Galicia is the Galician Public Foundation of Genomics Medicine (FPGMX) is a non-profit organization established to advance precision medicine in clinical practice and to ensure equitable access to genomic testing in public healthcare^7^. FPGMX works to the Galician Service of Health (SERGAS) providing comprehensive clinical genetics services to all hospitals, including both molecular and cytogenetic analyses, and covering approximately 2.7 million residents which are organized in seven Integrated Management Structures or Health Areas^7^ ^,^ ^8^ (Abad-Santos et al., 2024).

### 2.2 *DPYD* genotyping workflow: requests, analysis and turn-around-times

**FIGURE 1:**
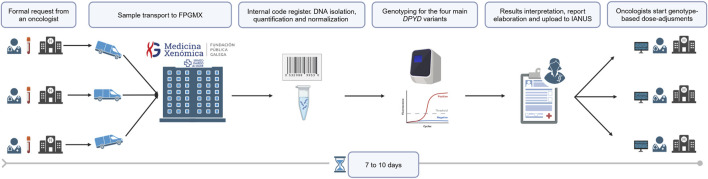
*DPYD* genotyping workflow in FPGMX. Created in BioRender.com.

The FPGMX daily receives requests from different hospitals in the Galician community through a daily internal transportation system (Abad-Santos et al., 2024). Requests for *DPYD* gene analysis are received from the different healthcare areas of Galicia, each of which has an average population of approximately 400,000 inhabitants. It is worth highlighting that the different care levels of the SERGAS work under a unified clinical electronic record system called IANUS, which consolidates all clinical information derived from the health activities of a citizen^9^ ^,^ ^10^. *DPYD* analysis requests are received from university hospital complexes and regional hospitals. Formal requests, either electronic or paper forms, including the reason for the request together with a blood sample in EDTA, are received from oncologists when requesting a *DPYD* analysis for a patient (Figure 1). Data from the following months of 2024 has been represented, although not analysed in order to illustrate a period of 3 years from EMA recommendations.

The main keywords included in the requests are: *TTO 5-FU, TTO 5-Fluorouracil, TTO Capecitabine, DPYD, DPD, Avoid Fluoropyrimidines toxicity*.

Upon arrival, blood samples are automatically assigned with an internal code to prevent external identification. Subsequently, DNA isolation, quantification, and normalization are carried out following the established commercial protocol^11^. Detection of *DPYD* variants is performed by using real-time PCR. Variants to be analyzed are those recommended in 2020 by regulatory agencies: rs3918290 (c.1905+1G>A), rs55886062 (c.1679T>G), rs56038477 (c.1236G>A) and rs67376798 (c.2846T>A). Report elaboration encompassing *DPYD* results, interpretation and subsequent recommendations, is performed by a genetics specialist in pharmacogenetic testing. The final *DPYD* report is uploaded to IANUS, allowing oncologists to establish dose adjustments based on each patient *DPYD* genotype.

In agreement with oncologists, feasible turn-around-times (TAT) have been established to incorporate *DPYD* testing into clinical practice without delaying routine. Thus, the request is sent to the FPGMX before initiating fluoropyrimidines treatment. Taking into consideration transport, registration and processing times, within a period of seven to ten calendar days, oncologists have already available the corresponding *DPYD* results in the platform IANUS. This determined TAT can be even less in case of clinical urgency. As a result, there exists a seamless exchange of information between oncologists and geneticists.

### 2.3 Ethical standards

The pharmacogenetic analyses were performed as a part of a routine clinical practice by FPGMX. Furthermore, the publication of this article has received approval from the Research Ethics Committee from Santiago-Lugo (CEI-SL) under code 2023/251.

## 3 Results

### 3.1 Number of *DPYD* requests

**FIGURE 2:**
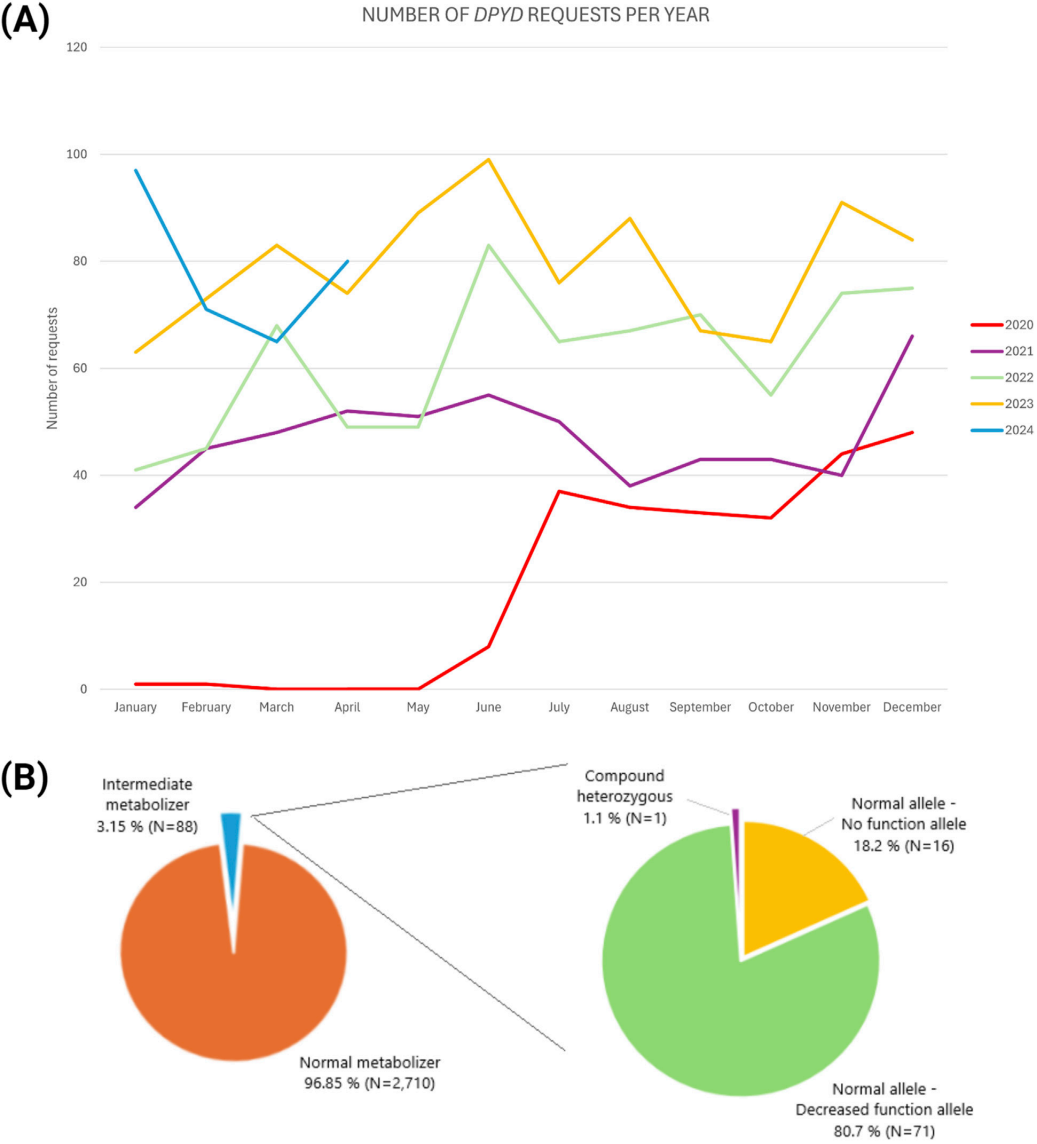
*DPYD* genotyping requests evolution from 2020 until April 2024 **(A)** and *DPYD* phenotype frequency for main variants in the Galician population **(B)** Created in BioRender.com.

A total of 2,798 *DPYD* requests received in the Pharmacogenetic Unit at the FPGMX have been analyzed during the period from 1st June 2020 to 1st May 2024, both included. Figure 2A illustrates the evolution in the number of *DPYD* requests per month. The rise in requests has progressed from a few samples before the change in recommendations in 2020 to 10–25 samples per week to the present, showcasing the impact of the EMA recommendation and the feasibility and utility of *DPYD* genotyping. It is noteworthy that the number of requests is still increasing, reaching significant values during June of 2023. In terms of percentages, the total number of requests has increased by 57.9% between 2021 and 2020, 23.8% between 2022 and 2021 and 22% between 2023 and 2022. The highest number was reached in June 2023, with ninety-night requests, followed by January 2024, with ninety-seven.

It is worth highlighting that prior 2020 the *DPYD* test was already optimized and ready-to-use at the Pharmacogenetics Unit, however the number of requests were very low with only fourteen cases from 2018 till April 2020. Additionally, those fourteen cases belong to patients that already developed toxicity to fluoropyrimidines treatment, additionally TAT were very short being between 24 and 48 h since the sample was received at FPGMX. It is worth highlighting that from these fourteen cases presenting toxicity to fluoropyrimidines, eleven were found to be wild type for the four study variants, one was categorized as wild type after analyzing only three variants (rs3918290, rs55886062 and rs67376798) and finally, two cases were heterozygous for the variant rs67376798.

### 3.2 Genotyping results

*DPYD* genotyping results performed in 2,798 Caucasian patients (1,633 male; 1,165 female) revealed that there is a 3.15% of heterozygosity for at least one of the four *DPYD* variants, a total of eighty-eight intermediate metabolizers for the *DPYD* gene. According to CPIC^®^ guidelines^12^, it is observed a phenotype distribution of 96.85% of normal metabolizers and 3.15% of intermediates metabolizers; in this line, normal metabolizer refers to a patient who do not present any of the four analyzed variants. From the intermediates, 80.7% were composed by a normal allele and a decreased function allele, the 18.2% were encompassed by a normal allele and a non-function allele, and finally, 1.1% were compound heterozygous. Specifically, sixteen presented a combination of a normal allele and a no function allele (either rs3918290 (c.1905+1G>A) or rs55886062 (c.1679T>G)), seventy-one samples presented a combination of a normal allele and decreased functional allele (either rs56038477 (c.1236G>A) or rs67376798 (c.2846A>T)), and one sample exhibited a compound heterozygote with two variants of reduced functionality (specifically rs56038477 + rs67376798 (c.1236G>A + c.2846A>T)). It is important to note that no homozygous genotypes have been found for any of the four variants of interest (Figure 2B).

In terms of polymorphisms frequency, the distribution observed for each risk variant was: 0.29% (N = 8) of samples with rs3918290 (c.1905+1G>A); 0.29% (N = 8) with rs55886062 (c.1679T>G); 1.31% (N = 37) presenting rs56038477 (c.1236G>A) and 1.20% (N = 34) with rs67376798 (c.2846T>A). The variant rs56038477 (c.1236G>A) resulted to be the most prevalent, while rs3918290 (c.1905+1G>A) was the least among the four polymorphisms observed.

## 4 Discussion

The publication of recommendations concerning *DPYD* analysis prior fluoropyrimidine derivatives prescription has resulted in the implementation of *DPYD* testing into clinical care from May 2020. Although in Galicia there was the possibility of analyzing *DPYD* variants prior 2020, the reality was that the requests were very limited. Furthermore, all those requests correspond to patients that have already developed adverse reactions, thus having a reactive nature (Henricks et al., 2019). From May 2020, the requests received at FPGMX are preventive, being performed before prescribing the medication, as regulatory agencies recommend. Thus, after May 2020 the number of samples were on the rise with 238 received until the end of the year, 565 samples during 2021, 741 in 2022 and 952 in 2023. It is noteworthy that this article describes the tendency of *DPYD* clinical routing request from a reference genomic medicine center in Galicia.

Another crucial aspect contributing to the accomplishment of *DPYD* integration in clinical care is the diligent pursuit by oncologists to identify the optimal approach for treating their patients with the feasible less toxicity. Additionally, the FPGMX has the capability to provide brief TAT for *DPYD* requests taking no longer than seven to ten calendar days. These values predict an upward trend in the forthcoming months with an increasing number of hospitals and oncologists requesting the test.

In terms of results, it is remarkable that, despite the low population frequency of the variants, from the 2,798 patients analyzed, 3.15% exhibited at least one variant of *DPYD*. A patient with one copy of a normal functional allele and either one copy of a no function allele or one copy of a decreased function allele is categorized as having an intermediate metabolizer (IM) phenotype. IMs for *DPYD* will exhibit reduced enzymatic activity with a high risk of developing toxicity to fluoropyrimidines (Lee et al., 2014; Henricks et al., 2018), thus it is necessary to establish dose-adjustments depending on each patient’s genotype. Clinical recommendations for IMs depend on the functionality of the carried mutated allele (non-function or decreased functional). Therefore, EMA and AEMPS recommendations suggest that IM patients should start treatment with a reduced dose^13^ ^,^ ^14^. Consortia such as CPIC^®^ and the DPWG also issue dose adjustments recommendations for IMs, and both recommend an initial dose reduction of 50% (Henricks et al., 2019; de With et al., 2023). Recent studies provide evidence to support recommendations of a 50% dose reduction in heterozygous patients (Henricks et al., 2018; Amstutz et al., 2018; Lunenburg et al., 2020).

A similar study carried out in Switzerland by Begré et al (2022) analyzed the impact of EMA recommendations on *DPYD* analysis within their diagnostic center. Authors assessed *DPYD* tests spanning the period from 2017 to 2020 and exposed the way changes in EMA recommendations have exerted an influence on the volume of conducted tests, with a noticeable increase being observed from June 2020. It is worth mentioning that when comparing the *DPYD* requests between the Swiss and Galician studies, those received before 2020 were reactive. Concerning the genotyping outcomes between both studies, the percentage of heterozygotes in the Swiss study was twice our percentage, with 6.3% and 3.15%, respectively. In addition, it should be noted that in our study no homozygotes were found for any of the variants analyzed; however, the Swiss study identified two homozygous, concretely for rs75017182 (c.1129-5923C>G) and for rs3918290 (c.1905+1G>A). Additionally, two compound heterozygotes were identified: one composed of rs75017182 + rs3918290 (c.1129–5923C>G/c.1905+1G>A) and the other of rs3918290 + rs67376798 (c.1905+1G>A/c.2846A>T). Our results indicate the presence of two heterozygotes, formed by rs56038477 + rs67376798 (c.1236G>A/c.2846A>T). In a similar study performed in Spain, the PhotoDPYD study, researchers observed a high frequency of *DPYD* gene variants. The study identified one individual with compound heterozygosity for rs3918290 + rs67376798 (c.1905+1G>A/c.2846A>T), two individuals with compound heterozygosity for rs75017182 + rs67376798 (c.1129–5923C>G/c.2846A>T), and one individual with compound heterozygosity for rs3918290 + rs75017182 (c.1905+1G>A/c.1129–5923C>G) (Miarons et al., 2023). In the same line, a smaller study highlights a high prevalence of loss-of-function variants in the dihydropyrimidine dehydrogenase gene and underscores the importance of genotyping these variants prior to initiating fluoropyrimidine treatment (Riera et al., 2021). Although *DPYD* testing is now mandatory, there are other recent studies supporting this importance (Wigle et al., 2021; Lau et al., 2023; Nguyen et al., 2024).

Another study evaluating the implementation of *DPYD* was published in 2023. The Working Group on the Implementation of DPD-deficiency Testing in Europe assessed the status of DPD testing before 2019, prior to the issuance of EMA recommendations, and after 2021. The study involved around hundred professionals across more than twenty countries. Results showed that following EMA recommendations most countries experienced a significant increase in genotype and phenotype testing, alongside the adoption of new local guidelines and increased test reimbursement in certain regions. In this manner, these tests transitioned from retrospective toxicity assessment to a proactive analysis aligned with EMA guidelines. Overall, EMA recommendations supported the implementation of DPD testing in Europe, emphasizing the importance of reimbursement and clear clinical guidelines for their success, while also highlighting the need to enhance oncologists’ awareness of the clinical significance of these tests in medical practice (de With et al., 2023).

Finally, it is noteworthy that out of the fourteen patients (data preceding 2020) who underwent DPYD analysis after developing ADRs, eleven did not exhibit any of the scrutinized variants. This fact highlights the importance of identifying novel polymorphisms that could potentially contribute to toxicity from 5-FU treatment in the case of European populations, such as c.1601G>A or c.299_302del (De Mattia et al., 2024; Pratt et al., 2024). Additionally, it is worth highlighting that CPIC variants are not necessarily adapted to all ethnicities, therefore depending the population, the inclusion of other pharmacogenetic variants already described in non-European ancestry populations, such as c.557A>G and c.868A>G (African ancestry) and c.2279C>T (South Asian ancestry) (Chan et al., 2024; Pratt et al., 2024), may be important in order to improve the pre-emptive management of severe adverse reactions in this type of patients.

It should be noted that our study has some limitations. On the one hand, this article highlights that 96.85% of the patients were categorized as normal metabolizers according to CPIC^®^ guidelines. However, as previously mentioned, absence of the analysed polymorphisms does not necessarily mean absence of toxicity. On the other hand, data related to patients’ follow-up is not available in this article. In this line, it is worth mentioning that on-going projects in collaboration with oncologists are considered the to analyze the outcome treatment and associated toxicities. Finally, until last year, rs56038477 (c.1236G>A) and rs75017182 (c.1129-5923C>G) were thought to be in perfect linkage disequilibrium (LD) and were used indistinctly for determining the presence of the HapB3 haplotype, in fact, AEMPs recommends the analysis of rs56038477 in its informative note delivered in May 2020 (García-Alfonso et al., 2022). However, recent findings suggest that rs56038477 may not always accurately reflect the HapB3 haplotype, which can lead to false-positives genotypes and suboptimal dose-adjustments (Turner et al., 2024).

Similarly to the rest of Europe, the incorporation of *DPYD* genotyping has experienced a notable increase in Galicia as a preliminary step to commencing fluoropyrimidine-based chemotherapy, considering the updated recommendations. While it has already been established as a clinical standard in several centers, with the support of the FPGMX, we expect a gradual rise in the volume of requests, ensuring comprehensive reach throughout Galicia.

## Data Availability

The data analyzed in this study is subject to the following licenses/restrictions: Data derived from clinical practice. Requests to access these datasets should be directed to Olalla Maroñas, olalla.maronas@usc.es.

## Footnotes

1 PharmGKB. Fluoropyrimidine Pathway, Pharmacokinetics. https://www.pharmgkb.org/pathway/PA150653776

2 PharmGKB Gene-specific Information Tables for DPYD https://www.pharmgkb.org/page/DPYDRefMaterials

3 European Medicines Agency (EMA). EMA recommendations on DPD testing prior to treatment with fluorouracil, capecitabine, tegafur and flucytosine (2020). https://www.ema.europa.eu/en/news/ema-recommendations-dpd-testing-prior-treatment-fluorouracil-capecitabine-tegafur-and-flucytosine

4 Agencia Española de Medicamentos y Productos Sanitarios. Fluorouracilo, capecitabina, tegafur y flucitosina en pacientes con déficit de dihidropirimidina deshidrogenasa (2020) https://www.aemps.gob.es/informa/fluorouracilo-capecitabina-tegafur-y-flucitosina-en-pacientes-con-deficit-de-dihidropirimidina-deshidrogenasa

5 Sociedad Española de Oncología Médica (SEOM). El cáncer en cifras. https://seom.org/prensa/el-cancer-en-cifras

6 Asociación Española Contra el Cáncer (AECC). AECC Observatorio. https://observatorio.contraelcancer.es/

7 Fundación Pública Galega de Medicina Xenómica. Inicio. https://xenomica.sergas.gal/?idioma=es

8 Consellería de Sanidade - Servizo Galego de Saúde. As áreas sanitarias e distritos sanitarios de Galicia. https://www.sergas.es/A-nosa-organizacion/As-sete-estruturas-de-Galicia?idioma=es

9 Diario Oficial de Galicia (DOG). DOG 34 del 18/02/2009 - DECRETO 29/2009, de 5 de febrero, por el que se regula el uso y acceso a la historia clíica electrónica. https://www.xunta.gal/dog/Publicados/2009/20090218/AnuncioA6C6_es.html

10 Consellería de Sanidade - Servizo Galego de Saúde. Consulta Historia Clínica y Farmaco–terapéutica. https://www.sergas.es/Asistencia-sanitaria/Consulta-Historia-Cl%C3%ADnica-e-Historial-Farmacoterap%C3%A9utico?idioma=es

11
https://assets.thermofisher.com/TFS-Assets/LSG/manuals/MAN0009593_TaqManSNP_UG.pdf

12 PharmGKB. CPIC® Allele Function and Phenotype. https://www.pharmgkb.org/page/cpicFuncPhen

13 European Medicines Agency (EMA). EMA recommendations on DPD testing prior to treatment with fluorouracil, capecitabine, tegafur and flucytosine (2020). https://www.ema.europa.eu/en/news/ema-recommendations-dpd-testing-prior-treatment-fluorouracil-capecitabine-tegafur-and-flucytosine

14 Agencia Española de Medicamentos y Productos Sanitarios. Fluorouracilo, capecitabina, tegafur y flucitosina en pacientes con déficit de dihidropirimidina deshidrogenasa (2020) https://www.aemps.gob.es/informa/fluorouracilo-capecitabina-tegafur-y-flucitosina-en-pacientes-con-deficit-de-dihidropirimidina-deshidrogenasa/

## Abbreviations

FPGMX, Galician Public Foundation of Genomics Medicine; AEMPS, Spanish Drug and Medical Devices Agency; TAT, Turn-around-times.
